# Endotracheal tube biofilm in critically ill patients during the COVID-19 pandemic : description of an underestimated microbiological compartment

**DOI:** 10.1038/s41598-022-26560-w

**Published:** 2022-12-27

**Authors:** Thomas Maldiney, Valentin Pineau, Catherine Neuwirth, Linda Ouzen, Isabelle Eberl, Géraldine Jeudy, Sophie Dalac, Lionel Piroth, Mathieu Blot, Marc Sautour, Frédéric Dalle, Caroline Abdulmalak, Romain Ter Schiphorst, Paul-Simon Pugliesi, Thomas Poussant, Agathe Ogier-Desserrey, Isabelle Fournel, Melchior de Giraud d’Agay, Marine Jacquier, Marie Labruyère, François Aptel, Jean-Baptiste Roudaut, Thibault Vieille, Pascal Andreu, Sébastien Prin, Pierre-Emmanuel Charles, Maël Hamet, Jean-Pierre Quenot

**Affiliations:** 1 Department of Intensive Care Medicine, William Morey General Hospital, Chalon-Sur-Saône, France; 2 Lipness Team, INSERM Research Centre LNC-UMR1231 and LabEx LipSTIC, University Bourgogne-Franche-Comté, Dijon, France; 3 Infectious Diseases Department, University Hospital of Dijon, Dijon, France; 4 Department of Bacteriology, University Hospital of Dijon, Dijon, France; 5 UMR/CNRS 6249 Chrono-Environnement, University Bourgogne Franche-Comté, Besançon, France; 6 Dermatology Department, University Hospital of Dijon, Dijon, France; 7 Centre d’Investigation Clinique, CHU Dijon; INSERM, CIC 1432, Module Epidémiologie Clinique, CHU Dijon, Dijon, France; 8 Department of Parasitology/Mycology, University Hospital of Dijon, Dijon, France; 9 UMR PAM A 02.102 Procédés Alimentaires et Microbiologiques, University Bourgogne Franche-Comté, Agrosup Dijon, Dijon, France; 10 Laboratoire de Biologie Médicale, William Morey General Hospital, Chalon-Sur-Saône, France; 11 Department of Intensive Care Medicine, University Hospital of Dijon, Dijon, France

**Keywords:** Microbiology, Medical research

## Abstract

Biofilm (BF) growth is believed to play a major role in the development of ventilator-associated pneumonia (VAP) in the intensive care unit. Despite concerted efforts to understand the potential implication of endotracheal tube (ETT)-BF dispersal, clinically relevant data are lacking to better characterize the impact of its mesostructure and microbiological singularity on the occurrence of VAP. We conducted a multicenter, retrospective observational study during the third wave of the COVID-19 pandemic, between March and May 2021. In total, 64 ETTs collected from 61 patients were included in the present BIOPAVIR study. Confocal microscopy acquisitions revealed two main morphological aspects of ETT-deposited BF: (1) a thin, continuous ribbon-shaped aspect, less likely monobacterial and predominantly associated with *Enterobacter spp*., *Streptococcus pneumoniae* or *Viridans* streptococci, and (2) a thicker, discontinuous, mushroom-shaped appearance, more likely characterized by the association of bacterial and fungal species in respiratory samples. The microbiological characterization of ETT-deposited BF found higher acquired resistance in more than 80% of analyzed BF phenotypes, compared to other colonization sites from the patient’s environment. These findings reveal BF as a singular microbiological compartment, and are of added clinical value, with a view to future ETT-deposited BF-based antimicrobial stewardship in critically ill patients.

*Trial registration* NCT04926493. Retrospectively registered 15 June 2021.

## Introduction

Ventilator-associated pneumonia (VAP) is the most frequent healthcare-associated infection (HAI) in the intensive care unit (ICU)^1^. It is not only one of the most critical risk factors associated with significant morbidity and mortality in critically ill patients^2^, but is also responsible for a major increase in overall hospital length of stay (LOS) and associated crude costs^3^. VAP treatment relies on early and appropriate antimicrobial therapy in order to limit the emergence of multidrug-resistant (MDR) species^4,5^. Several preventive measures have been described in the literature to reduce the incidence of VAP and mitigate its clinical impact in the ICU^6,7^. Among these, preventing biofilm (BF) formation on the inner surface of the endotracheal tube (ETT) appears to hold promise, and has garnered increasing attention in recent years^8,9^.

BF is commonly defined as a well-structured assembly of both bacterial and fungal elements embedded in a self-produced multifaceted organic polysaccharide matrix^10^. Its development on medical devices, notably ETT, is thought to be responsible for the occurrence of VAP through several mechanisms, from dispersion to aerosolization^11^. In addition, BF extension might also lead to significant ETT obstruction with a direct impact on mechanical ventilation (MV)^12^. Despite concerted efforts to better apprehend the underlying conditions associated with BF architecture^8^, formation^13,14^, staging^15^ and microbiological characterization^16^, there is a marked lack of relevant clinical data documenting a causal relationship between the development of BF and VAP, which could serve as a basis for BF-directed prevention or subsequent treatment^17^.

The present BIOPAVIR (Characterization of ETT-deposited BIOfilm in view of future VAP prevention in Intensive Care Unit) study aimed to investigate the development of BF on ETTs, and to describe the mesostructural and microbiological characterization of ETT-deposited BF in critically ill patients at increased risk for the development of VAP in the ICU during the third wave of the COVID-19 pandemic. The underlying hypothesis was that we would identify a robust multimodal BF characterization, with a view to future interventional studies evaluating the impact of BF removal and ETT cleaning through close-suctioning systems^12,18,19^.

## Methods

### Study characteristics, patients and data collection

We collected ETTs for routine microbiological analysis from all patients who were admitted to the two participating ICUs and who required invasive MV during the third wave of the COVID-19 pandemic in France (March 1 to May 31, 2021). Two ICUs participated in the study, namely one ICU from a general (non-academic) hospital (William Morey Hospital, Chalon-sur-Saône, France) and one medical ICU from a university teaching hospital (University Hospital of Dijon, Dijon, France). Patients over 18 years of age, admitted to the ICU and requiring MV for more than 48 h were included in the study. Patients whose ETT could not be properly collected and dispatched for microbiological analysis and confocal microscopy within 24–48 h were excluded from the study.

At extubation, each ETT was cleaned three times with a sterile saline solution to remove any excess of tracheal secretion (inner and outer surfaces), and was then immediately placed in a sterile bottle, and dispatched to the microbiological laboratory for further analysis, according to a local biofilm protocol (see “Microbiological characterization” section below). All ETTs were cut into four sterile sections, approximately 0.5 cm thick (two below-the-cuff sections and two above-the-cuff sections) for further mesostructural and microbiological analysis.

Data were retrospectively collected using a dedicated electronic Case Report Form (eCRF) (CleanWeb®), completed by the investigators in each study center. For all eligible patients, we recorded the following demographic and clinical information regarding the ICU stay: age, gender, type of admission, COVID-19 disease status (confirmed by PCR), number of organ dysfunction and severity of the disease, medical history, anthropometric reference data, duration of MV, length of ICU stay, occurrence of reintubation, ICU mortality and occurrence of VAP. The eCRF also recorded the structural characteristics (shape, thickness) and microbiological data related to the characterization of ETT-deposited BF, as well as a global microbiological colonization cartography for each patient (respiratory samples from tracheobronchial aspirate (TBA) or bronchoalveolar lavage (BAL), blood cultures from a central venous catheter (CVC) or arterial catheter (AC), urine analysis, and other colonization/infection sites).

### Study definitions

Both William Morey Hospital and University Hospital of Dijon adapted their prevention policies to be in line with that described in the guidelines of the French Society of Anesthesia and Intensive Care Medicine and the French Society of Intensive Care on healthcare-associated pneumonia in the ICU^21^. VAP diagnosis was confirmed by the physician directly in charge of the patient, after a minimum duration of MV of 48 h, according to the same guidelines.

Immunosuppression was defined as immunosuppressive therapy, long-term corticosteroid therapy (> 3 months), Human Immunodeficiency Virus (HIV) infection, solid organ and bone marrow transplants.

Prior antimicrobial treatment referred to any antimicrobial treatment from among amoxicillin/clavulanic acid, quinolone, second and third generation cephalosporins, during the 3 months prior to ICU admission.

Commensal oropharyngeal flora (COF) were considered for each sample, retrieving the association of more than two bacterial species among classical anaerobic (like *Peptostreptococcus* species) and aerobic species (Viridans streptococci, Staphylococcus spp., Streptococcus spp., Haemophilus spp., Neisseria spp., Corynebacterium spp., …) from normal oropharyngeal flora.

### Mesostructural characterization

Adapted from a recent method developed by Thorarinsdottir et al. for the processing of ETT before electron microscopy analysis^22^, half of the ETT sections (below- and above-the-cuff sections dedicated to confocal microscopy) were fixed in a solution of 4% formaldehyde diluted with phosphate-buffered saline (PBS) for 30 min at room temperature, then washed in normal saline solution to remove the excess formaldehyde with gentle shaking for 10 min. All ETT sections were then allowed to dry at room temperature for a minimum of 72 h before confocal microscopy acquisitions.

Confocal imaging was achieved with a VivaScope®3000 system from Caliber Imaging and Diagnostics (Rochester, NY, USA). Briefly, ETT below- and above-the-cuff sections were placed under the handheld optical lens of the VivaScope®3000 system to provide up to ten [750 µm × 750 µm]-wide image z-stacks covering the whole inner surface of the ETT. Each image z-stack included between ten and forty separate acquisitions by an optical pace of 5 µm. Given its large field of view, the VivaScope®3000 gives access to high resolution imaging of the biofilm structure at the mesoscopic scale (approximately within 1 mm), as defined in the work by Wagner et al.^23^. Table S1 introduces the main characteristics and differences between both the VivaScope®3000 and a classical Leica Confocal Laser Scanning Microscopy (CLSM) system.

Quantitative confocal image analysis and ETT-deposited BF thickness were accessible with a contrast-based ImageJ 1.52o protocol (Plot Profile Function) and calculated as the mean of three representative measurements throughout each ETT section. ETT-deposited BF mesostructural acquisitions and analysis were performed by a physician who was blinded to all patient information based on three qualitative morphological criteria: surface aspect (smooth or rugged), attachment to the inner surface of the ETT (detached or tied) and inner covering of the ETT (continuous or intermittent). Ribbon-shaped BF were described as smooth, detached, and continuous. Mushroom-shaped BF were both rugged and intermittently tied to the inner surface of the ETT.

### Microbiological characterization

The remaining half of the ETT sections (below- and above-the-cuff sections) were gently homogenized in a 20% Digest-EUR® solution (Eurobio Scientific, Les Ulis, France) for 15 min at 37 °C according to a previously described protocol^16,24^. After a few seconds of gentle shaking, an aliquot of the solution was then spread on different culture plates (trypticase soy agar, blood agar under aerobic and anaerobic atmospheres, Drigalski agar, chocolate agar with the addition of 5% CO_2_, and Sabouraud agar) incubated for 48 h at 37 °C, either pure or at 1/10 dilution. Subsequent BF bacterial counts were determined according to laboratory protocols used for respiratory samples. Bacterial identification was done using matrix-assisted laser desorption-ionization-time of flight mass spectrometry (MALDI-TOF MS, Bruker Daltonics, Germany) and susceptibility testing was performed using standard clinical routine methods according to the latest CASFM / EUCAST guidelines^25^.

For each patient, microbiological BF characterization was compared to all respiratory samples (TBA-BAL) and global colonization sites (TBA-BAL, CVC-AC blood cultures, urine analysis as well as other colonization / infection sites) available during the whole ICU stay.

### Statistical analysis

GraphPad Prism version 5 was used for statistical analysis. Results are displayed as number (percentage, %) for categorical variables and as median (interquartile range, IQR) for quantitative variables. The Chi-square test was used to compare qualitative variables, and the Mann–Whitney test for quantitative variables after verification of the normality of distribution. Pearson’s correlation coefficient (*r)* was calculated to evaluate the strength of the linear correlation between ETT-deposited BF thickness and MV duration. All *p* values were two-tailed and differences were considered as significant for *p* values < 0.05 (* for *p* values between 0.01 and 0.05 ; ** for *p* values between 0.001 and 0.01 ; *** for *p* values < 0.001 ; ns stands for non significant).

### Ethics approval and consent to participate

In line with current French legislation governing clinical research, this study falls outside the scope of Jardé’s law^20^, and as such, the Ethics Committee CPP (Comité de Protection des Personnes) Est I confirmed its approval for all experimental methods and informed consent was not required. The study protocol was conducted in accordance with the relevant guidelines and regulations. Patients were nonetheless informed in the admission booklet and via posters in the ICU that the data from their medical files could be used retrospectively for research purposes.

## Results

### Study and patient characteristics

**Figure 1:**
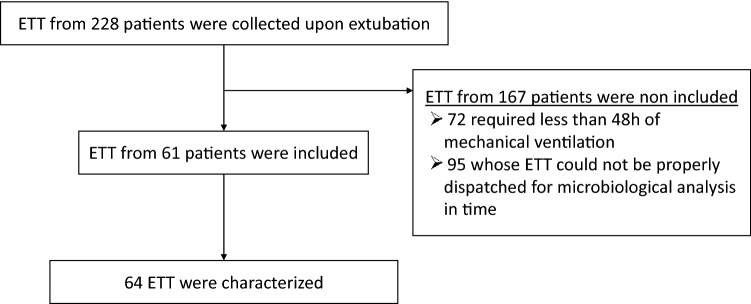
Flow chart for study screening and inclusion of ETT collection.

We collected ETTs from a total of 228 patients in the ICU between March and May 2021. As described in the study flowchart (Fig. 1), 61 patients were retrospectively included in the BIOPAVIR study, corresponding to a total of 64 ETTs (Table S2). ETTs from 167 patients were not included from the study due to a duration of MV < 48 h (72 patients) or inability to properly dispatch ETT for subsequent analysis (95 patients).

Table 1 presents the main characteristics of the study population at ICU admission for all collected ETT. The median (IQR) age was 69 years (30–88), with similar proportions of men and women, and a median body mass index (BMI) within the overweight range at 29 kg/m^2^ (17–54). COVID-19 acute respiratory distress syndrome (ARDS) accounted for a large majority of the ICU medical admissions, with a total of 40 patients (67%). High blood pressure (31%), diabetes (18%) and dyslipidemia (18%) were the most frequent chronic diseases in the study population. The median length of hospital stay before ICU admission was 2 days (0–83) and approximately one quarter of patients received prior antimicrobial treatment. The median simplified acute physiology score (SAPS) II was 43 (13–92) and the median Sepsis-related Organ Failure Assessment (SOFA) score was 6 (2–19). The median duration of MV was 10 days (3–62), with a reintubation rate at 25% (15 patients) and the median length of ICU stay was 15 days (3–73). VAP occurred in 32 patients (52%) with a median Clinical Pulmonary Infection Score (CPIS) of 7 (4–10).

**Table 1:** Characteristics of study patients from ETT collection.

	Total (n = 61)
**Age**	69 (30–88)
**Male gender**	33 (54)
**Medical admission**	58 (97)
**ICU admission for ARDS**	45 (74)
**COVID-19**	40 (67)
**SAPS II**	43 (13–92)
**SOFA**	6 (2–19)
**Chronic diseases**	
High blood pressure	31 (51)
Diabetes	18 (30)
Dyslipidemia	18 (30)
Chronic respiratory disease	6 (10)
COPD	3 (5)
Ischemic cardiomyopathy	7 (11)
Cirrhosis	4 (7)
Chronic kidney disease	5 (8)
Immunosuppression	8 (13)
**Length of hospital stay before ICU admission**	2 (0–83)
**BMI**	29 (17–54)
**Prior antimicrobial treatment**	16 (26)
**VAP**	32 (52)
CPIS	7 (4–10)
**ICU stay**	
Total duration of MV	10 (3–62)
Reintubation	15 (25)
Length of ICU stay	15 (3–73)
ICU mortality	20 (33)

All results are displayed as number (%) for categorical variables and as median (IQR) for quantitative variables.

ARDS acute respiratory distress syndrome, BMI body mass index, COPD chronic obstructive pulmonary disease, CPIS clinical pulmonary infection score, MV mechanical ventilation, ns not significant, SAPS simplified acute physiology score, SOFA sepsis-related organ failure assessment, VAP ventilator-associated pneumonia.

### Mesostructural characterization

**Figure 2:**
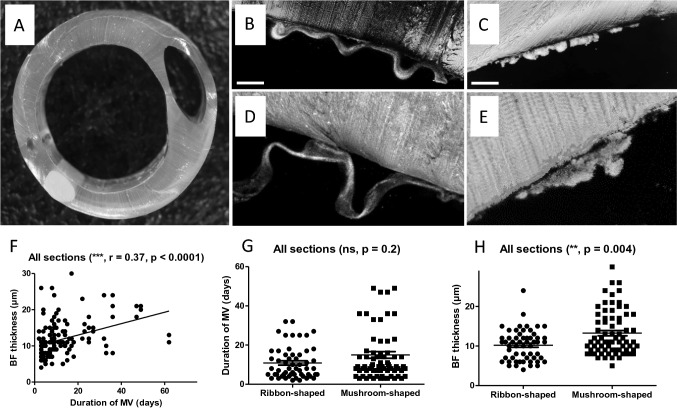
Mesostructural characterization of ETT-deposited BF. (**A**) Macroscopic view of ETT section before confocal microscopy ; (**B**) confocal image of an ETT section showing ribbon-shaped BF deposited on the inner surface (scale bar, 75 µm) ; (**C**) confocal image of an ETT section showing mushroom-shaped BF deposited on the inner surface (scale bar, 75 µm) ; (**D**) zoomed confocal image of an ETT section showing ribbon-shaped BF deposited on the inner surface (scale bar, 25 µm) ; (**E**) zoomed confocal image of an ETT section showing mushroom-shaped BF deposited on the inner surface (scale bar, 25 µm) ; (**F**) correlation curve between the length of MV duration and BF thickness ; (**G**) comparison of median MV duration between ribbon- and mushroom-shaped BF ; H. comparison of median BF thickness between ribbon- and mushroom-shaped BF. BF biofilm, ETT endotracheal tube, MV mechanical ventilation, ns not significant.

Figure 2 details the mesostructural characterization of ETT-deposited BF based on confocal imaging of all ETT sections. Figure 2A shows a typical macroscopic view of a 0.5 cm-thick ETT section before confocal imaging. Figure 2B–E reveal two microscopic BF aspects. The first, ribbon-shaped BF aspect, is displayed in Fig. 2B and D (n = 28). It appears to form a thin, continuous serpentine band that physically detaches from the inner surface of the ETT section. The second, mushroom-shaped BF aspect, displayed in Fig. 2C and E, constitutes a discontinuous, eruptive formation strictly attached to the inner surface of the ETT section (n = 36). Despite the absence of significant correlation, BF was detected on all ETT and overall quantitative analysis of ETT mesostructural characterization only found a weak correlation between BF thickness and total MV duration (Pearson’s r coefficient 0.37, *p* < 0.0001, Fig. 2F). Out of the 64 collected ETT, only 3 displayed both ribbon and mushroom morphologies within the same ETT. However, given the preparation and analysis of multiple ETT sections (below- and above-the-cuff sections, for a total of six ETT sections), and the preeminence of one morphology in all cases (> 4 ETT sections displaying the same morphology), results displayed in the present manuscript return the information associated with the predominant morphology for each sample. Considering all patients and ETT sections, the mushroom-shaped BF aspect was slightly predominant (56%). Although the morphological aspect of ETT-deposited BF did not seem to be associated with the duration of MV (Fig. 2G), ribbon-shaped BF were significantly thinner than mushroom-shaped BF, as demonstrated in Fig. 2H. As shown in Supplementary Fig. S1A to S1F, results were similar in both below-the-cuff and above-the-cuff sections, with thinner ribbon-shaped BF in comparison to mushroom-shaped BF. Mesostructural characterization of ETT-deposited BF depending on the occurrence of VAP and COVID-19 is shown in Fig. S2. The occurrence of VAP was significantly associated with increased BF thickness (Fig. S2A), while COVID-19 status had no impact on BF thickness (Fig. S2B). Data from Fig. S2C to S2E confirm that BF type (ribbon-shaped or mushroom-shaped) was not dependent on the occurrence of VAP, COVID-19 or the Gram-staining of the retrieved bacteria (Gram-negative or Gram-positive bacteria).

### Microbiological characterization

**Figure 3:**
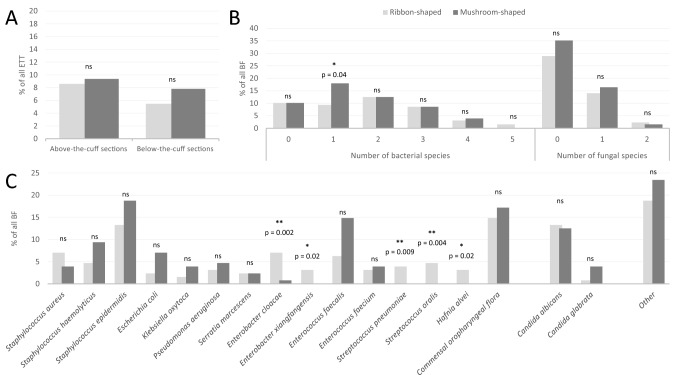
Microbiological characterization of ribbon- and mushroom-shaped ETT-deposited BF. (**A**) compared percentage of ETT retrieving both bacterial and fungal species between ribbon- and mushroom-shaped ETT-deposited BF ; (**B**) compared number of both bacterial and fungal species between ribbon- and mushroom-shaped ETT-deposited BF ; (**C**) compared distribution of both bacterial and fungal species between ribbon- and mushroom-shaped ETT-deposited BF. BF biofilm, ETT endotracheal tube, ns not significant. Other (microbiological species retrieved < 5%) : *Candida kefyr*, *Candida lusitaniae*, *Candida parapsilosis*, *Candida tropicalis*, *Citrobacter freundii, Enterobacter cancerogenus, Enterococcus durans, Klebsiella aerogenes*, *Klebsiella variicola*, *Lactobacillus gasseri, Lactobacillus paracasei, Lactobacillus rhamnosus*, *Morganella morganii, Neisseria mucosa, Propionibacterium acnes, Proteus mirabilis, Pseudomonas oryzihabitans*, *Raoultella ornithinolytica, Serratia rubidae, Staphylococcus hominis, Stenotrophomonas maltophilia.*

The comparison of the microbiological characterization of ribbon- and mushroom-shaped ETT-deposited BF is presented in Fig. 3. Overall, 30% of ETT-deposited BFs had both bacterial and fungal species, with no significant difference in proportions according to BF shape or localization of the section (below-the-cuff or above-the-cuff) (Fig. 3A). There was a larger proportion of monobacterial BF among mushroom-shaped BF, as compared to ribbon-shaped BF (Fig. 3B). Otherwise, the number of both bacterial and fungal species was largely similar in both ribbon- and mushroom-shaped ETT-deposited BF, notably regarding the number of fungal species and bacterial associations composed of two and more species. A more detailed analysis of the distribution of bacterial and fungal species within the BF is presented in Fig. 3C. *Staphylococcus epidermidis*, *Enterococcus faecalis*, *Staphylococcus haemolyticus*, *Staphylococcus aureus*, *Escherichia coli*, *Pseudomonas aeruginosa* and *Enterobacter cloacae* all feature among the most frequent bacterial species composing the ETT-deposited BF. *Candida albicans* and *Candida glabrata* were the predominant fungal species, with a larger proportion of the former. A similar microbiological distribution was confirmed when comparing below-the-cuff with above-the-cuff ETT sections (Fig. S3). Finally, *Enterobacter cloacae*, *Enterobacter xiangfangensis*, *Streptococcus pneumoniae*, *Steptococcus oralis* and *Hafnia alvei* were predominantly associated with the formation of ribbon-shaped BF on ETT.

**Figure 4:**
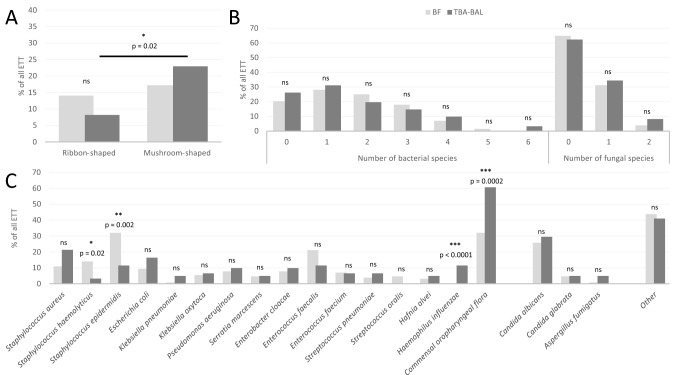
Microbiological characterization of ETT-deposited BF in comparison to respiratory samples (TBA-BAL). (**A**) compared percentage of ETT retrieving both bacterial and fungal species between ETT-deposited BF and respiratory samples; (**B**) compared number of both bacterial and fungal species between ETT-deposited BF and respiratory samples ; (**C**) compared distribution of both bacterial and fungal species between ETT-deposited BF and respiratory samples. BAL bronchoalveolar lavage, BF biofilm, ETT endotracheal tube, ns not significant, TBA tracheobronchial aspirate. Other (microbiological species retrieved < 5%) : *Candida kefyr*, *Candida lusitaniae*, *Candida parapsilosis*, *Candida tropicalis*, *Citrobacter freundii, Enterobacter cancerogenus, Enterococcus durans, Klebsiella aerogenes*, *Klebsiella variicola*, *Lactobacillus gasseri, Lactobacillus paracasei, Lactobacillus rhamnosus*, *Morganella morganii, Neisseria mucosa, Propionibacterium acnes, Proteus mirabilis, Pseudomonas oryzihabitans*, *Raoultella ornithinolytica, Serratia rubidae, Staphylococcus hominis, Stenotrophomonas maltophilia.*

The comparison of microbiological characterization obtained from ETT-deposited BF and from respiratory samples is presented in Fig. 4 and Fig. S4. The formation of ribbon-shaped biofilm on ETT was correlated with a smaller proportion of associations between bacterial and fungal species in respiratory samples (Fig. 4A). The number of bacterial and fungal species was highly comparable in ETT-deposited BF and respiratory samples (Fig. 4B). Although respiratory samples and BF appeared to follow the same distribution profile regarding fungal species (Fig. 4C), with a larger proportion of *Candida albicans* in both cases, two points deserve to be noted: firstly, the identification of coagulase-negative staphylococci (CoNS) was strongly associated with the development of BF on ETT; and secondly, both *Haemophilus influenzae* and COF were more commonly retrieved in respiratory samples than in BF (Fig. S4) Fig. S5 displays the microbiological characterization of ETT-deposited BF depending on the occurrence of VAP and COVID-19. Apart from *Serratia marcescens,* which was never retrieved in BF of patients without VAP, the distribution of bacterial and fungal species within the BF was independent of the occurrence of VAP (Fig. S5A). Results from Fig. S5B show a different trend with *Staphylococcus epidermidis*, *Enterococcus faecalis* and COF, more likely to be retrieved in BF of patients who tested positive for COVID-19.

**Figure 5:**
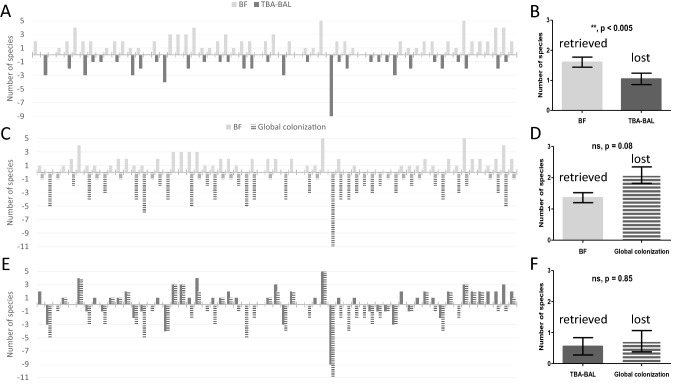
Microbiological species either retrieved or lost from BF analysis in comparison to respiratory samples (TBA-BAL) or global colonization (TBA-BAL, CVC-AC, urinalysis and other colonization/infection sites). (**A**) number of microbiological species either retrieved or lost from BF analysis in comparison to respiratory samples for each patient ; (**B**) absolute value of the median number of microbiological species either retrieved or lost from BF analysis in comparison to respiratory samples, all patients combined ; (**C**) number of microbiological species either retrieved or lost from BF analysis in comparison to global colonization for each patient ; (**D**) absolute value of the median number of microbiological species either retrieved or lost from BF analysis in comparison to global colonization, all patients combined ; (**E**) lost and found differential for BF microbiological species in comparison to respiratory samples or global colonization for each patient ; (**F**) absolute value of the median lost and found differential for BF microbiological species in comparison to respiratory samples or global colonization, all patients combined. AC arterial catheter, BAL bronchoalveolar lavage, BF biofilm, CVC central veinous catheter, TBA tracheobronchial aspirate.

Figure 5 presents the global cartography of the microbiological species either retrieved (stated as a positive value in the histogram) or lost (stated as a negative value in the histogram) in BF when compared to respiratory samples or overall patient colonization. Despite the loss of significant species identified in respiratory samples only, microbiological characterization of ETT-deposited BF enabled the identification of multiple microbiological species that were never retrieved from the respiratory samples in a large majority of the study patients (Fig. 5A). Figure 5B shows the absolute median value of the number of species found only in BF or in respiratory samples. All patients considered, two species are retrieved in BF, as compared to only one bacterial species lost from respiratory samples. Similar information regarding the comparison of BF and global colonization are provided in Fig. 5C and D. Although several species appear to be lost from global colonization, Fig. 5D illustrates that microbiological characterization of BF enables the identification of supplemental species. The difference between lost and found BF species, in comparison to either respiratory samples or global colonization, is presented in Fig. 5E. Positive values on the histogram demonstrate the ability of BF microbiological characterization to identify species that are not found in either respiratory samples or global patient colonization. A synthesized view of the BF cartography of the microbiological species is summarized in Fig. 5F, presenting the median differential gain of one species in BF compared to respiratory samples, but also the corresponding loss of one species from BF in favour of global patient colonization.

Among the 61 patients included in the BIOPAVIR study, the comparison of antibiotic resistance phenotypes between ETT-deposited BF and other colonization/infection sites was available for 35 patients (57%), corresponding to a total of 50 bacterial species tested for discordant phenotypes. Table 2 shows the list of 11 discordant phenotypes identified, with the most frequent resistance mechanism associated with their expression. Overall, more than 80% of these discordant phenotypes demonstrate a higher resistance profile in BF than in either respiratory samples or other colonization/infection sites, with the most preeminent species being *Staphylococcus aureus*, *Escherichia coli*, *Pseudomonas aeruginosa* and *Stenotrophomonas maltophilia*.

**Table 2:** Comparison of discordant antibiotic resistance phenotypes between ETT-deposited BF and colonization/infection sites for eleven patients.

Patient	Bacterial species	MIC in BF (mg/L)	Resistance phenotype in BF	MIC in colonization site (mg/L)	Discordant phenotype from colonization sites	Most suspected resistance mechanism	Highest resistance in BF
1	*Stenotrophomonas maltophilia*	> 16	TMP/SMX-resistant	0,125	TMP/SMX-susceptible (TBA-BAL)	Acquisition of *sul* genes	+
6	*Staphylococcus aureus*	32 (Benzylpenicillin)	Penicillinase	0,06	Wild-type (TBA-BAL)	Acquisition of *blaZ* genes	+
12	*Escherichia coli*	32 (Piperacillin/tazobactam)	High-level penicillinase	< 4	Low-level penicillinase (urinalysis)	Acquisition of *blaTEM* genes	+
13	*Escherichia coli*	16 (Ampicillin)	Low-level penicillinase	2	Wild-type (blood culture)	Hyperproduction of the acquired penicillinase	+
14	*Stenotrophomonas maltophilia*	24	Ceftazidime-resistant	4	Wild-type (TBA-BAL)	Hyperproduction of the chromosomal β-lactamase (L2)	+
21	*Staphylococcus haemolyticus*	< 0,03	Rifampin-susceptible	> 2	Rifampin-resistant (blood culture)	Mutation in *rpoB* genes	–
26	*Pseudomonas aeruginosa*	8	Gentamicin-resistant	3	Gentamicin-susceptible (TBA-BAL)	Acquisition of *aac* genes	+
27	*Klebsiella aerogenes*	16 (ceftazidime)	High-level cephalosporinase	< 1	Wild-type (TBA-BAL)	Hyperproduction of the chromosomal cephalosporinase (AmpC)	+
36	*Staphylococcus aureus*	> 4	Erythromycin-resistant	1	Erythromycin-susceptible (TBA-BAL)	Acquisition of *erm* genes	+
49	*Raoultella ornithinolytica*	1 (Amoxicillin/clavulanic acid)	Wild-type	32	High-level penicillinase (TBA-BAL)	Hyperproduction of the chromosomal penicillinase	–
57	*Pseudomonas aeruginosa*	> 128	Ceftazidime-resistant	1	Wild-type (TBA-BAL)	Hyperproduction of the chromosomal cephalosporinase (AmpC)	+

BAL bronchoalveolar lavage, BF biofilm, TBA tracheobronchial aspirate, TMP/SMX trimethoprim/sulfamethoxazole.

## Discussion

This study provides complementary insights into the structural and microbiological characterization of ETT-deposited BF as a potential underestimated, singular and individualized compartment in critically ill patients during the third wave of the COVID-19 pandemic. First, we identify the formation of two different BF aspects using confocal microscopy, and a tendency towards increased BF thickness with the total length of MV duration. Then, despite a possible signature species of each BF profile, the description of the microbiological cartography associated with ETT-deposited BF enables the identification of supplemental species, as compared to the usual global patient colonization. Finally, the comparison of antibiotic resistance phenotypes between ETT-deposited BF and other colonization/infection sites shows a higher resistance profile in BF for the predominant species usually associated with VAP.

### Study and patient characteristics

Briefly, our patients’ characteristics illustrate a clinical profile that is similar to that described in the recent study by Blonz et al. regarding the epidemiology and microbiology of VAP in COVID-19 patients, notably the age, predominant chronic diseases, and median SAPS II and SOFA scores^26^. In accordance with the recent identification of obesity as major risk factor for COVID-19 mortality, the median BMI of the population was found to be close to 30 kg/m^2^ ^27^. Despite the absence of a clear consensus, and multiple discussions questioning the routine clinical use of a Clinical Pulmonary Infection Score (CPIS) score > 6 for the diagnosis of VAP^28,29^, our data confirm a median CPIS score of 7 in patients with VAP. The length of ICU stay as well as the occurrence of VAP also largely reflect the findings of the multicenter retrospective study by Blonz et al., with a long duration of MV and ICU stay, a significant reintubation rate due to the higher risk of developing VAP, and a mortality rate close to 30%^26^.

### Mesostructural characterization

The morphological information provided by the analysis of ETT-deposited BF by confocal microscopy identified two mesoscopic BF profiles for the first time. Contrary to CLSM, which allows ultra-high resolution imaging and the detection of complex microscopic structures within the biofilm (the microscopic scale being defined around 100 µm), the VivaScope®3000 system–similar to what optical coherence tomography (OCT) would permit–gives access to high resolution imaging of the biofilm structure at the mesoscopic scale, i.e. approximately within a 1 mm range (Table S1). Indeed, Wagner et al.^23^, but also Morgenroth and Milferstedt^30^, demonstrated the critical importance of structural properties of the mesoscale to understand the ecological mechanisms associated with the development of biofilm structure^30^. Previous results using optical coherence tomography^14,31^ and confocal microscopy^32^ from both human and animal in vivo clinical trials as well as in vitro studies seem to have already identified morphological aspects similar to the one described here as mushroom-shaped BF. However, the ribbon-shaped BF displayed in Fig. 2B and D appears smooth and thinner, detaching from the inner surface of the ETT section. Interestingly, these images resemble some of those obtained from scanning electron microscopy reported by Torres et al.^32,33^. In line with the recent study by Thorarinsdottir et al., comparing BF formation on different ETT surfaces or materials^22^, we found no impact of the ICU on the preferential morphology of ETT-deposited BF. Despite the absence of significant differences in the present study, there is an overall tendency towards the preeminence of ribbon-shaped BF for MV of shorter duration (Fig. 2G). Despite the inability to visualize the aspect of ETT-deposited BF in real time throughout the MV of each patient, the results from the present study seem to corroborate the progressive formation of BF during MV, with a weak but significant correlation between BF thickness and the duration of MV. These data were confirmed for ETT-deposited BF from either below- or above-the-cuff and may possibly reflect two successive stages in the formation of biofilm. Still, their interpretation should be qualified due to the necessary fixation step of ETT sections following extubation, with a possible direct impact on, and modification of the BF structure during such chemical fixation. Consequently, these two biofilm aspects warrant confirmation in a larger cohort before envisioning any generalization to critically ill patients requiring mechanical ventilation for more than 48 h. Besides, additional clinical studies reporting both microscopic (with CLSM) and mesoscopic (with the VivaScope®3000 or an OCT system) information would be essential and complementary to better understand the impact of ETT-biofilm development on the occurrence of VAP.

### Microbiological characterization

Regarding the method employed for the dislodgement of bacterial biofilms, the use of cultures after sonication was demonstrated to be superior for orthopedic devices^34^. However, the ESCMID Biofilm guidelines published in 2015 clearly stated that regarding patients with ETT biofilm, “mucus from within the ETT can be aspirated and cultured to identify ETT pathogens in ETT biofilms that may have caused VAP^35^”. Moreover, our group recently demonstrated a clear superiority of the chemical treatment dithiothreitol (Digest-EUR) over sonication for the dislodgement of *Staphylococcus epidermidis* biofilm on ETT^24^. Based on both our recent experience and the ESCMID Biofilm guidelines, the present work used a mucus reagent (Digest-EUR) for the microbiological processing of biofilm. Additional work and microbiological studies comparing multiple methods and pathogens to provide a complete view of the best protocol and the most efficient dislodgement of bacterial biofilms on endotracheal tube would be required in the future.

Compared to previous important studies reporting the microbiological characterization of ETT-deposited BF^16,36,37^, the present BIOPAVIR study provides additional data comparing the microbiological composition of biofilms from ETT sections from below- and above-the-cuff, with the global colonization retrieved in ICU patients via the usual respiratory samples^33,38^. Despite a lower proportion of *Pseudomonas aeruginosa* and a significant proportion of negative cultures, the distribution of bacterial and microbiological species illustrated in Fig. 3C is largely comparable to that described by Danin et al.^16^ or Vandecandelaere et al.^36^, with a large predominance of Coagulase-negative staphylococci (CoNS), *Enterococcus faecalis*, *Staphylococcus aureus*, *Enterobacter cloacae* and *Candida albicans*. Regarding the comparison of microbiological composition of ETT-deposited BF and respiratory samples (Fig. 4C), our results differ from those reported by de Ferreira et al. on biofilms recovered from ETT in pediatric ICU patients showing a comparable distribution of microbiological species^37^. Notably, in the present BIOPAVIR study, the identification of CoNS occurred more frequently in ETT-deposited BF than in respiratory samples, even more so in patients with COVID-19, while *Haemophilus influenzae* and COF are more likely retrieved in respiratory samples. We believe this difference could result from the longer MV duration during the third wave of the COVID-19 pandemic, as compared to the MV duration reported in pediatric patients. Interestingly, the small proportion of COF retrieved in BF compared to respiratory samples argues strongly for the quality of ETT section preparation and the reliable nature of the microbiological information extracted from BF.

Despite an overall tendency towards increased BF thickness with the occurrence of VAP, data from the present study should be analyzed with caution due to the small number of ETT per microbiological species, and this precludes confirmation of Wilsons’ hypothesis regarding the preferential occurrence of VAP with advanced biofilm stage^15^ or the discovery of a reliable association between microbiological species and any BF aspect. Yet, these preliminary results open potential new perspectives towards an enhanced understanding of the development of ETT-deposited BF in the ICU. To the best of our knowledge, our results (notably Fig. 5) displays one of the first attempts to compare the microbiological cartography of all species retrieved in BF to that gathered from global colonization of ICU patients during the third wave of the COVID-19 pandemic, including the usual respiratory samples as well as CVC and AC blood cultures, urinalysis, stool, drainage exudates and any other relevant colonization/infection site as decided by the attending physician. The main information to be retained from these histograms is the identification of ETT-deposited BF as an underestimated microbiological compartment, independently of all other colonization/infection sites. These results demonstrate the ability of BF analysis to retrieve additional species, which may sometimes go unnoticed from all other colonization sites. Along with the data from Table 2 listing discordant antibiotic resistance phenotypes retrieved in BF, these results show the potential added value and importance of complete microbiological characterization of BF plus global colonization from ICU patients requiring MV. Indeed, these analyses provided precious information with perspectives for future BF-based antimicrobial stewardship, especially considering that more than 80% of analyzed discordant bacterial phenotypes from BF acquired significant antibiotic resistance mechanisms, with the subsequent risk of developing VAP with multidrug-resistant pathogens.

Despite being a multicenter study with quite a large sample size and homogeneous groups when compared to similar previous works focusing on the development of BF on ETT, this study nevertheless has some limitations. First, microbiological characterization would benefit from the addition of molecular-based techniques such as 16S/18S analyses of the BF microbiota to provide additional and complementary information regarding the composition of BF, when compared to the present culture-based only data. Apart from prior antimicrobial treatment listed in the patient characteristics, data related to antimicrobial therapy during the ICU stay were not recovered in the present study. Given the large panel of microbiological pathogens identified as potentially responsible for the development of VAP or other infectious complications, and also given the large number of clinical or microbiological variables already considered, any attempt to analyze the potential impact of antimicrobial therapy or interplay between these therapies and the biofilm structure would have resulted in a failure to highlight any relevant tendency. A more detailed analysis of the impact of antimicrobial treatment would require specific focus on a single bacterial species, such as in the study by Fernández-Barat et al. describing the impact of both linezolid and vancomycin on the composition of endotracheal tube *Staphylococcus aureus* biofilms from ICU patients^38^. Consequently, other clinical studies describing the impact of antimicrobial treatments would be essential to better understand the impact of such molecules on the development and composition of ETT biofilms. In addition, further prospective studies in larger cohorts are warranted to confirm the results described in the present BIOPAVIR study, notably regarding the microbiological data and given the relatively small number of patients associated with each species. Such restrictions hamper convenient translation of the present results into information regarding a possible association between the formation of BF and subsequent occurrence of VAP. However, we believe that the routine clinical use of confocal microscopy with complete microbiological overview dedicated to BF characterization at the mesoscopic scale not only constitutes a major strength of the present work, but also reveals ETT-deposited BF to be an underestimated and specific microbiological compartment with possible future implications for antimicrobial stewardship in critically ill patients.

## Conclusions

The BIOPAVIR study reports additional morphological and microbiological data regarding ETT biofilm characterization and formation in ICU patients requiring MV for more than 48 h during the third wave of the COVID-19 pandemic. In addition to the confocal microscopy-based identification of a double morphological BF aspect on ETT, each associated with a specific microbiological signature and resistance phenotype, the results of the present BIOPAVIR study support the idea that ETT-deposited BF might be considered as an underestimated and independent microbiological compartment, separately from all other colonization sites. Accordingly, the development of BF sampling as well as microbiological phenotypic cartography could be envisioned with a view to improved antimicrobial stewardship, most importantly in critically ill patients at increased risk for the development of VAP.

## Supplementary Information

Supplementary Information.

## Data Availability

The data analyzed during the BIOPAVIR study are available from the corresponding author on reasonable request.

## Abbreviations

AC: Arterial catheter
ARDS: Acute respiratory distress syndrome
BAL: Bronchoalveolar lavage
BF: Biofilm
BMI: Body mass index
CoNS: Coagulase-negative staphylococci
COF: Commensal oropharyngeal flora
COPD: Chronic obstructive pulmonary disease
CPIS: Clinical pulmonary infection score
CVC: Central veinous catheter
eCRF: Electronic Case Report Form
ETT: Endotracheal tube
HAI: Healthcare-associated infection
HAP: Healthcare-acquired pneumonia
HIV: Human immunodeficiency virus
ICU: Intensive care unit
IQR: Interquartile range
LOS: Lengths of stay
ns: Not significant
MDR: Multidrug-resistant
MV: Mechanical ventilation
PBS: Phosphate-buffered saline
SAPS: Simplified acute physiology score
SOFA: Sepsis-related organ failure assessment
TBA: Tracheobronchial aspirate
VAP: Ventilator-associated pneumonia
