# Supplementary material for: Endotracheal tube biofilm in critically ill patients during the COVID-19 pandemic : description of an underestimated microbiological compartment
Source: Sci Rep. 2022 Dec 27;12:22389. doi: 10.1038/s41598-022-26560-w (PMC9794690; doi:10.1038/s41598-022-26560-w)
Supplement: Supplementary file 1 — Supplementary Information. [file 41598_2022_26560_MOESM1_ESM.docx]

**Endotracheal tube biofilm in critically ill patients during the COVID-19 pandemic : description of an underestimated microbiological compartment**

Thomas Maldiney^1,2^, Valentin Pineau^3^, Catherine Neuwirth^4,5^, Linda Ouzen^4^, Isabelle Eberl^3^, Géraldine Jeudy^6^, Sophie Dalac^6^, Lionel Piroth^3,7^, Mathieu Blot^2,3^, Marc Sautour^8^, Frédéric Dalle^8,9^, Caroline Abdulmalak^1^, Romain Ter Schiphorst^1^, Paul-Simon Pugliesi^1^, Thomas Poussant^1^, Agathe Ogier-Desserrey^10^, Isabelle Fournel^7^, Melchior de Giraud d’Agay^7^, Marine Jacquier^11^, Marie Labruyère^11^, François Aptel^11^, Jean-Baptiste Roudaut^11^, Thibault Vieille^11^, Pascal Andreu^11^, Sébastien Prin^11^, Pierre-Emmanuel Charles^2,11^, Maël Hamet^1^, Jean-Pierre Quenot^2,7,11^

^1^Department of Intensive Care Medicine, William Morey General Hospital, Chalon-sur-Saône, France.

^2^Laboratoire Lipness, UMR 1231, INSERM, Université de Bourgogne-Franche-Comté, Dijon, France.

^3^Infectious Diseases Department, University Hospital of Dijon, Dijon, France.

^4^Department of Bacteriology, University Hospital of Dijon, Dijon, France.

^5^UMR/CNRS 6249 Chrono-environnement, University of Bourgogne-Franche-Comté, Besançon, France.

^6^Dermatology Department, University Hospital of Dijon, Dijon, France.

^7^Inserm Centre d'Investigation Clinique (CIC) 1432, Module plurithématique, University of Burgundy, Dijon, France

^8^Laboratoire de Parasitologie-Mycologie, Plateforme de Biologie Hospitalo-Universitaire Gérard Mack, Dijon, France.

^9^Univ. Bourgogne Franche-Comté, Agrosup Dijon, UMR PAM A 02.102, Dijon, France.

^10^Laboratoire de Biologie Médicale, William Morey General Hospital, Chalon-sur-Saône, France.

^11^Department of Intensive Care Medicine, University Hospital of Dijon, Dijon, France.

***Table S1.*** Compared characteristics of the VivaScope®3000 and a Leica CLSM (Confocal Laser Scanning Microscopy) system

|  | **VivaScope3000** | **Leica TCS SP5 laser scanning confocal system** |
| --- | --- | --- |
| **Characteristics** | imaging wavelength at 830 nm, viewable section of 750 μm x 750 μm, resolution at 1024 x 1024 pixels, 6 frames per second | UV, Vis and IR in one system, Excitation range 350–1050 nm, up to 64 Megapixels/image, up to 250 frames/s, Line frequencies from 1Hz to 16 KHz |
| **Pros** | **handheld microscope**, practical and **easy to use for on-site clinical applications**, live biofilms imaging, unlimited max. mapped field, 30 x water immersion objective with high optical resolution (horizontal ~ 1.25 μm in center of image field, vertical ~ 5 μm in center of image field), adapted for **mesoscopic structures** | reproducible biofilm culturing format, live biofilms imaging, **antibody- and lectin-conjugated dyes can be used to quantitatively study biofilm matrix**, resolution up to 8k x 8k per image with up to 250 frames per second, multiple imaging wavelengths, **ultra-high optical vertical / horizontal resolution** (<< 1 µm), adapted for **microscopic structures** |
| **Cons** | no conjugated dye, resolution at 1024 x 1024 pixels, 6 frames per second, only **one imaging wavelength**, not adapted for microscopic structures | **non-portable system**, difficult to transpose to routine clinical application, not adapted for mesoscopic structures |
| **References** | - Braghiroli NF et al. The skin through reflectance confocal microscopy - Historical background, technical principles, and its correlation with histopathology. An Bras Dermatol. 2022 Nov-Dec;97(6):697-703 - https://www.vivascope.de/products/vivascope1500-3000/ | - Reichhardt C and Parsek MR. Confocal Laser Scanning Microscopy for Analysis of Pseudomonas aeruginosa Biofilm Architecture and Matrix Localization. Front Microbiol. 2019 Apr 2;10:677 - https://www.leica-microsystems.com/products/confocal-microscopes/p/leica-tcs-sp5/ |

***Table S2.*** List of the 64 ETT with COVID-19 status, duration of MV, biofilm type and median thickness

| ***ETT number*** | ***Patient number*** | ***Participating ICU*** | ***COVID-19*** | ***Duration of MV per ETT in days*** | ***Biofilm type*** | ***Median biofilm thickness in µm (interquartile range, IQR)*** |
| --- | --- | --- | --- | --- | --- | --- |
| ***1*** | ***1*** | ***University Hospital of Dijon*** | ***+*** | ***4*** | ***Mushroom-shaped*** | ***16 (15-17)*** |
| ***2*** | ***2*** | ***University Hospital of Dijon*** | ***+*** | ***3*** | ***Mushroom-shaped*** | ***19 (10-24)*** |
| ***3*** | ***3*** | ***University Hospital of Dijon*** | ***+*** | ***5*** | ***Ribbon-shaped*** | ***6 (5-9)*** |
| ***4*** | ***4*** | ***University Hospital of Dijon*** | ***+*** | ***7*** | ***Ribbon-shaped*** | ***14 (12-17)*** |
| ***5*** | ***5*** | ***University Hospital of Dijon*** | ***-*** | ***7*** | ***Mushroom-shaped*** | ***9 (9-10)*** |
| ***6*** | ***6*** | ***University Hospital of Dijon*** | ***+*** | ***4*** | ***Mushroom-shaped*** | ***8 (6-10)*** |
| ***7*** | ***7*** | ***University Hospital of Dijon*** | ***+*** | ***49*** | ***Mushroom-shaped*** | ***20 (18-24)*** |
| ***8*** | ***8*** | ***University Hospital of Dijon*** | ***-*** | ***32*** | ***Ribbon-shaped*** | ***17 (12-22)*** |
| ***9*** | ***9*** | ***University Hospital of Dijon*** | ***+*** | ***33*** | ***Mushroom-shaped*** | ***9 (8-12)*** |
| ***10*** | ***10*** | ***William Morey Hospital*** | ***+*** | ***14*** | ***Mushroom-shaped*** | ***10 (9-11)*** |
| ***11*** | ***11*** | ***William Morey Hospital*** | ***-*** | ***14*** | ***Mushroom-shaped*** | ***11 (9-12)*** |
| ***12*** | ***12a*** | ***William Morey Hospital*** | ***+*** | ***13*** | ***Ribbon-shaped*** | ***5 (4-7)*** |
| ***13*** | ***12b*** | ***William Morey Hospital*** | ***+*** | ***7*** | ***Mushroom-shaped*** | ***9 (7-11)*** |
| ***14*** | ***13*** | ***University Hospital of Dijon*** | ***-*** | ***36*** | ***Mushroom-shaped*** | ***18 (17-20)*** |
| ***15*** | ***14*** | ***University Hospital of Dijon*** | ***-*** | ***22*** | ***Mushroom-shaped*** | ***13 (11-24)*** |
| ***16*** | ***15*** | ***William Morey Hospital*** | ***-*** | ***8*** | ***Mushroom-shaped*** | ***15 (12-26)*** |
| ***17*** | ***16*** | ***University Hospital of Dijon*** | ***+*** | ***11*** | ***Mushroom-shaped*** | ***20 (18-20)*** |
| ***18*** | ***17*** | ***William Morey Hospital*** | ***-*** | ***8*** | ***Mushroom-shaped*** | ***9 (7-11)*** |
| ***19*** | ***18*** | ***William Morey Hospital*** | ***+*** | ***8*** | ***Ribbon-shaped*** | ***6 (5-7)*** |
| ***20*** | ***19*** | ***William Morey Hospital*** | ***+*** | ***5*** | ***Ribbon-shaped*** | ***14 (11-15)*** |
| ***21*** | ***20a*** | ***University Hospital of Dijon*** | ***+*** | ***4*** | ***Ribbon-shaped*** | ***11 (9-14)*** |
| ***22*** | ***20b*** | ***University Hospital of Dijon*** | ***+*** | ***2*** | ***Ribbon-shaped*** | ***13 (11-17)*** |
| ***23*** | ***21*** | ***University Hospital of Dijon*** | ***+*** | ***25*** | ***Ribbon-shaped*** | ***14 (11-15)*** |
| ***24*** | ***22*** | ***University Hospital of Dijon*** | ***-*** | ***5*** | ***Ribbon-shaped*** | ***9 (7-12)*** |
| ***25*** | ***23*** | ***University Hospital of Dijon*** | ***-*** | ***3*** | ***Ribbon-shaped*** | ***7 (6-8)*** |
| ***26*** | ***24*** | ***University Hospital of Dijon*** | ***+*** | ***4*** | ***Mushroom-shaped*** | ***11 (10-12)*** |
| ***27*** | ***25*** | ***William Morey Hospital*** | ***-*** | ***12*** | ***Ribbon-shaped*** | ***11 (10-12)*** |
| ***28*** | ***26*** | ***University Hospital of Dijon*** | ***+*** | ***18*** | ***Ribbon-shaped*** | ***8 (7-11)*** |
| ***29*** | ***27*** | ***University Hospital of Dijon*** | ***+*** | ***11*** | ***Ribbon-shaped*** | ***12 (11-13)*** |
| ***30*** | ***28*** | ***University Hospital of Dijon*** | ***-*** | ***9*** | ***Mushroom-shaped*** | ***14 (12-16)*** |
| ***31*** | ***29*** | ***William Morey Hospital*** | ***-*** | ***15*** | ***Ribbon-shaped*** | ***7 (6-8)*** |
| ***32*** | ***30*** | ***University Hospital of Dijon*** | ***+*** | ***36*** | ***Mushroom-shaped*** | ***12 (8-17)*** |
| ***33*** | ***31*** | ***William Morey Hospital*** | ***+*** | ***62*** | ***Mushroom-shaped*** | ***13 (9-14)*** |
| ***34*** | ***32a*** | ***University Hospital of Dijon*** | ***+*** | ***27*** | ***Ribbon-shaped*** | ***13 (12-17)*** |
| ***35*** | ***32b*** | ***University Hospital of Dijon*** | ***+*** | ***9*** | ***Mushroom-shaped*** | ***17 (15-18)*** |
| ***36*** | ***33*** | ***William Morey Hospital*** | ***+*** | ***4*** | ***Ribbon-shaped*** | ***6 (5-7)*** |
| ***37*** | ***34*** | ***University Hospital of Dijon*** | ***-*** | ***3*** | ***Mushroom-shaped*** | ***8 (7-9)*** |
| ***38*** | ***35*** | ***William Morey Hospital*** | ***+*** | ***16*** | ***Mushroom-shaped*** | ***10 (9-12)*** |
| ***39*** | ***36*** | ***University Hospital of Dijon*** | ***-*** | ***3*** | ***Ribbon-shaped*** | ***6 (5-9)*** |
| ***40*** | ***37*** | ***William Morey Hospital*** | ***-*** | ***6*** | ***Ribbon-shaped*** | ***7 (6-9)*** |
| ***41*** | ***38*** | ***William Morey Hospital*** | ***-*** | ***8*** | ***Mushroom-shaped*** | ***13 (9-14)*** |
| ***42*** | ***39*** | ***University Hospital of Dijon*** | ***+*** | ***9*** | ***Mushroom-shaped*** | ***12 (10-12)*** |
| ***43*** | ***40*** | ***William Morey Hospital*** | ***+*** | ***47*** | ***Mushroom-shaped*** | ***21 (19-22)*** |
| ***44*** | ***41*** | ***University Hospital of Dijon*** | ***+*** | ***10*** | ***Mushroom-shaped*** | ***10 (8-21)*** |
| ***45*** | ***42*** | ***University Hospital of Dijon*** | ***+*** | ***17*** | ***Mushroom-shaped*** | ***11 (10-25)*** |
| ***46*** | ***43*** | ***William Morey Hospital*** | ***-*** | ***23*** | ***Mushroom-shaped*** | ***16 (14-17)*** |
| ***47*** | ***44*** | ***William Morey Hospital*** | ***-*** | ***14*** | ***Mushroom-shaped*** | ***15 (14-18)*** |
| ***48*** | ***45*** | ***University Hospital of Dijon*** | ***-*** | ***8*** | ***Mushroom-shaped*** | ***8 (7-9)*** |
| ***49*** | ***46*** | ***University Hospital of Dijon*** | ***+*** | ***17*** | ***Ribbon-shaped*** | ***12 (10-14)*** |
| ***50*** | ***47*** | ***William Morey Hospital*** | ***+*** | ***7*** | ***Mushroom-shaped*** | ***8 (7-10)*** |
| ***51*** | ***48*** | ***University Hospital of Dijon*** | ***-*** | ***7*** | ***Mushroom-shaped*** | ***9 (7-10)*** |
| ***52*** | ***49*** | ***University Hospital of Dijon*** | ***+*** | ***15*** | ***Ribbon-shaped*** | ***13 (11-13)*** |
| ***53*** | ***50*** | ***University Hospital of Dijon*** | ***+*** | ***10*** | ***Ribbon-shaped*** | ***12 (9-14)*** |
| ***54*** | ***51*** | ***University Hospital of Dijon*** | ***+*** | ***3*** | ***Ribbon-shaped*** | ***5 (4-5)*** |
| ***55*** | ***52*** | ***University Hospital of Dijon*** | ***+*** | ***13*** | ***Ribbon-shaped*** | ***12 (11-14)*** |
| ***56*** | ***53*** | ***William Morey Hospital*** | ***+*** | ***9*** | ***Mushroom-shaped*** | ***18 (12-23)*** |
| ***57*** | ***54*** | ***University Hospital of Dijon*** | ***+*** | ***3*** | ***Mushroom-shaped*** | ***9 (7-9)*** |
| ***58*** | ***55*** | ***William Morey Hospital*** | ***+*** | ***8*** | ***Ribbon-shaped*** | ***13 (11-14)*** |
| ***59*** | ***56*** | ***University Hospital of Dijon*** | ***+*** | ***9*** | ***Mushroom-shaped*** | ***10 (8-10)*** |
| ***60*** | ***57*** | ***University Hospital of Dijon*** | ***+*** | ***3*** | ***Mushroom-shaped*** | ***10 (8-11)*** |
| ***61*** | ***58*** | ***University Hospital of Dijon*** | ***+*** | ***5*** | ***Ribbon-shaped*** | ***6 (5-7)*** |
| ***62*** | ***59*** | ***University Hospital of Dijon*** | ***+*** | ***25*** | ***Ribbon-shaped*** | ***14 (12-18)*** |
| ***63*** | ***60*** | ***University Hospital of Dijon*** | ***+*** | ***5*** | ***Ribbon-shaped*** | ***6 (5-7)*** |
| ***64*** | ***61*** | ***University Hospital of Dijon*** | ***+*** | ***4*** | ***Mushroom-shaped*** | ***9 (7-21)*** |

***Figure S1***. Mesostructural characterization of ETT-deposited BF


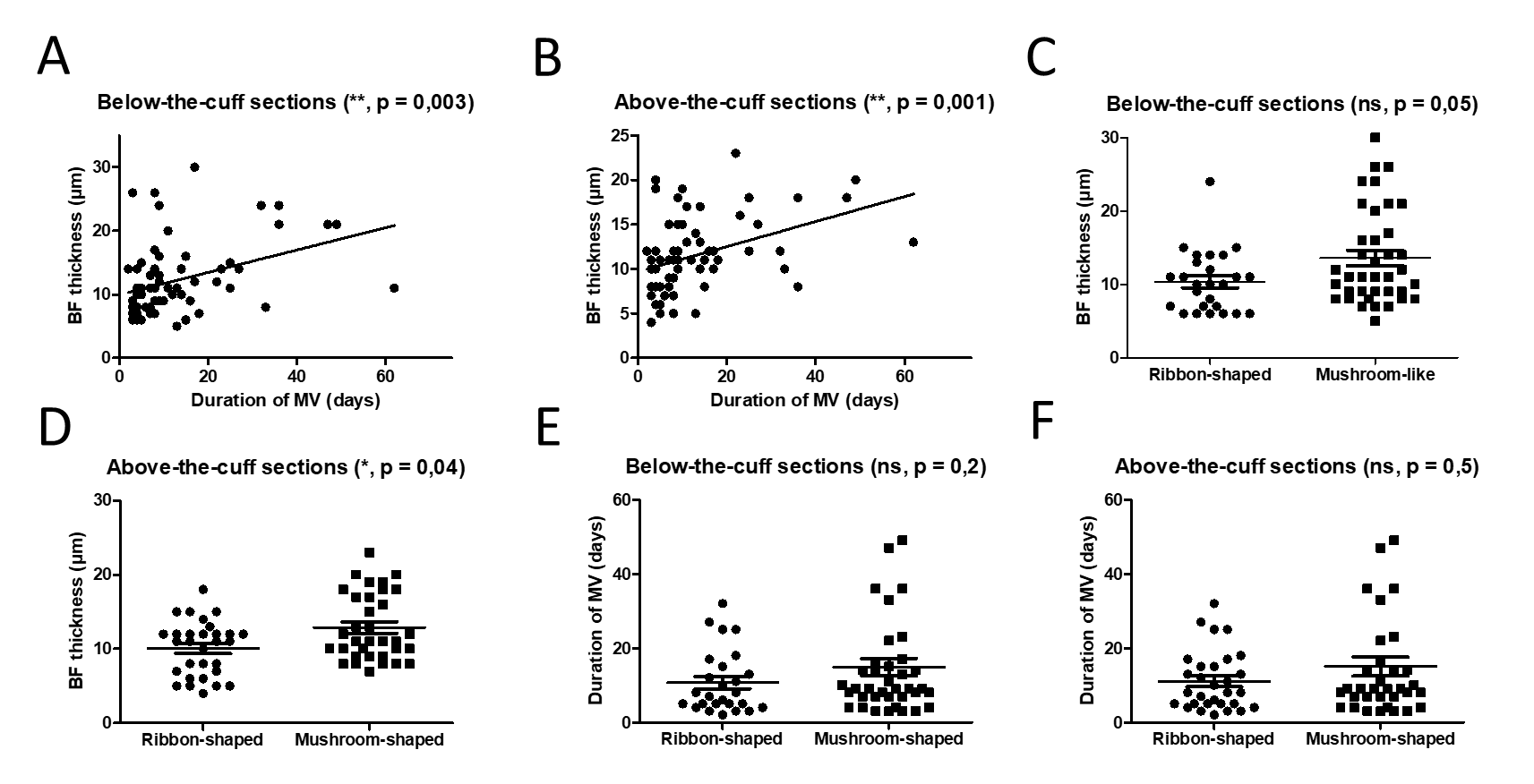


A. correlation curve between the length of MV duration and BF thickness for below-the-cuff sections ; B. correlation curve between the length of MV duration and BF thickness for above-the-cuff sections ; C. comparison of BF thickness between patients with ribbon- and mushroom-shaped BF from below-the-cuff sections analysis ; D. comparison of BF thickness between patients with ribbon- and mushroom-shaped BF from above-the-cuff sections analysis ; E. comparison of MV duration between patients with ribbon- and mushroom-shaped BF from below-the-cuff sections analysis ; F. comparison of MV duration between patients with ribbon- and mushroom-shaped BF from above-the-cuff sections analysis.

BF biofilm, ETT endotracheal tube, MV mechanical ventilation, ns not significant

***Figure S2***. Mesostructural characterization of ETT-deposited BF depending on the occurrence of VAP and COVID-19


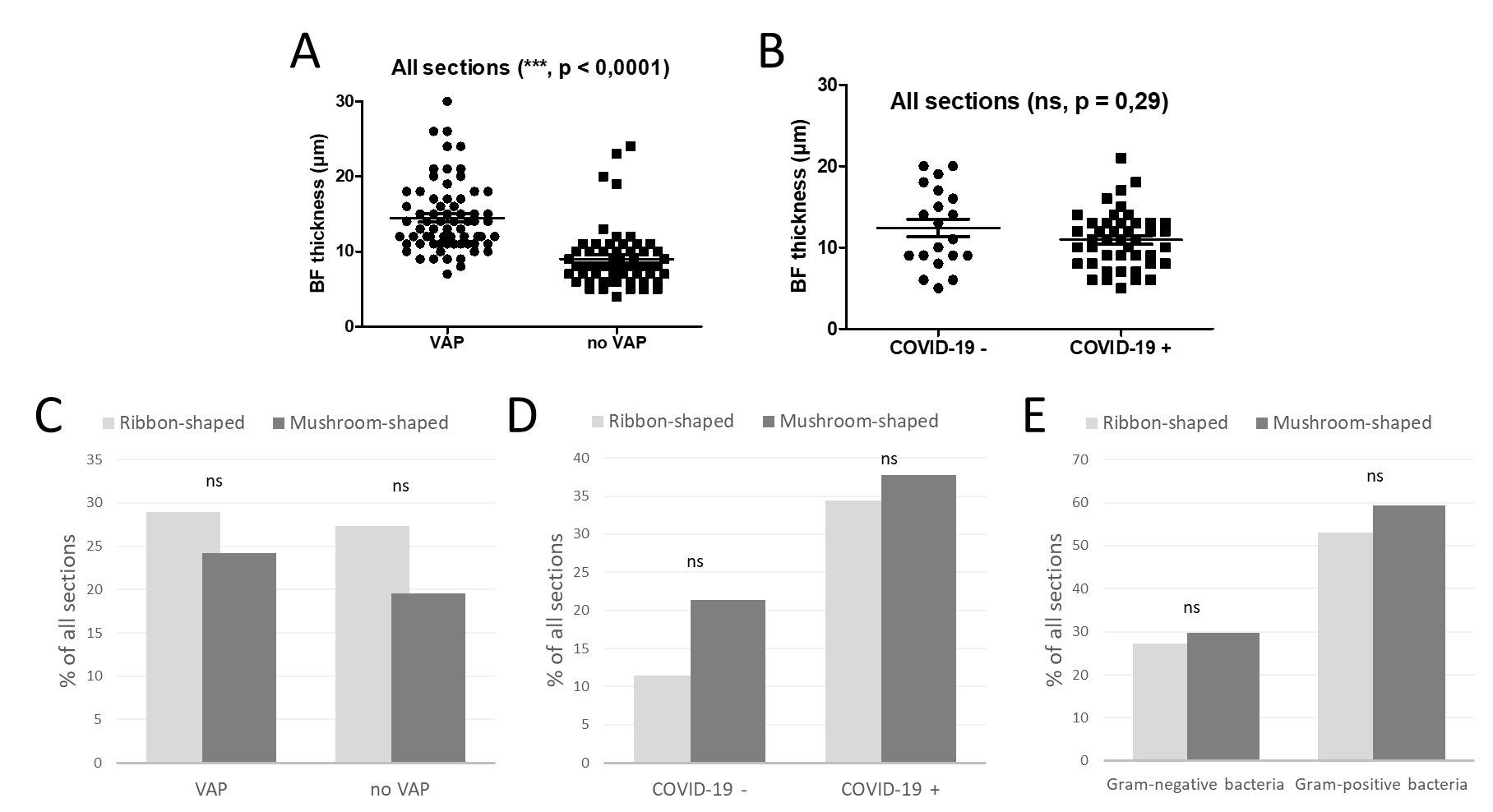


A. comparison of median BF thickness depending on the occurrence of VAP ; B. comparison of median BF thickness between patients with COVID-19 and those without COVID-19 ; C. compared percentage of ribbon- and mushroom-shaped BF depending on the occurrence of VAP ; D. compared percentage of ribbon- and mushroom-shaped BF depending on the occurrence of COVID-19 ; E. compared percentage of ribbon- and mushroom-shaped BF between Gram-negative and Gram-positive bacteria.

BF biofilm, ETT endotracheal tube, ns not significant, ns not significant, VAP ventilator-associated pneumonia

***Figure S3***. Compared distribution of both bacterial and fungal species between above-the-cuff sections and below-the-cuff sections analysis of ETT-deposited BF


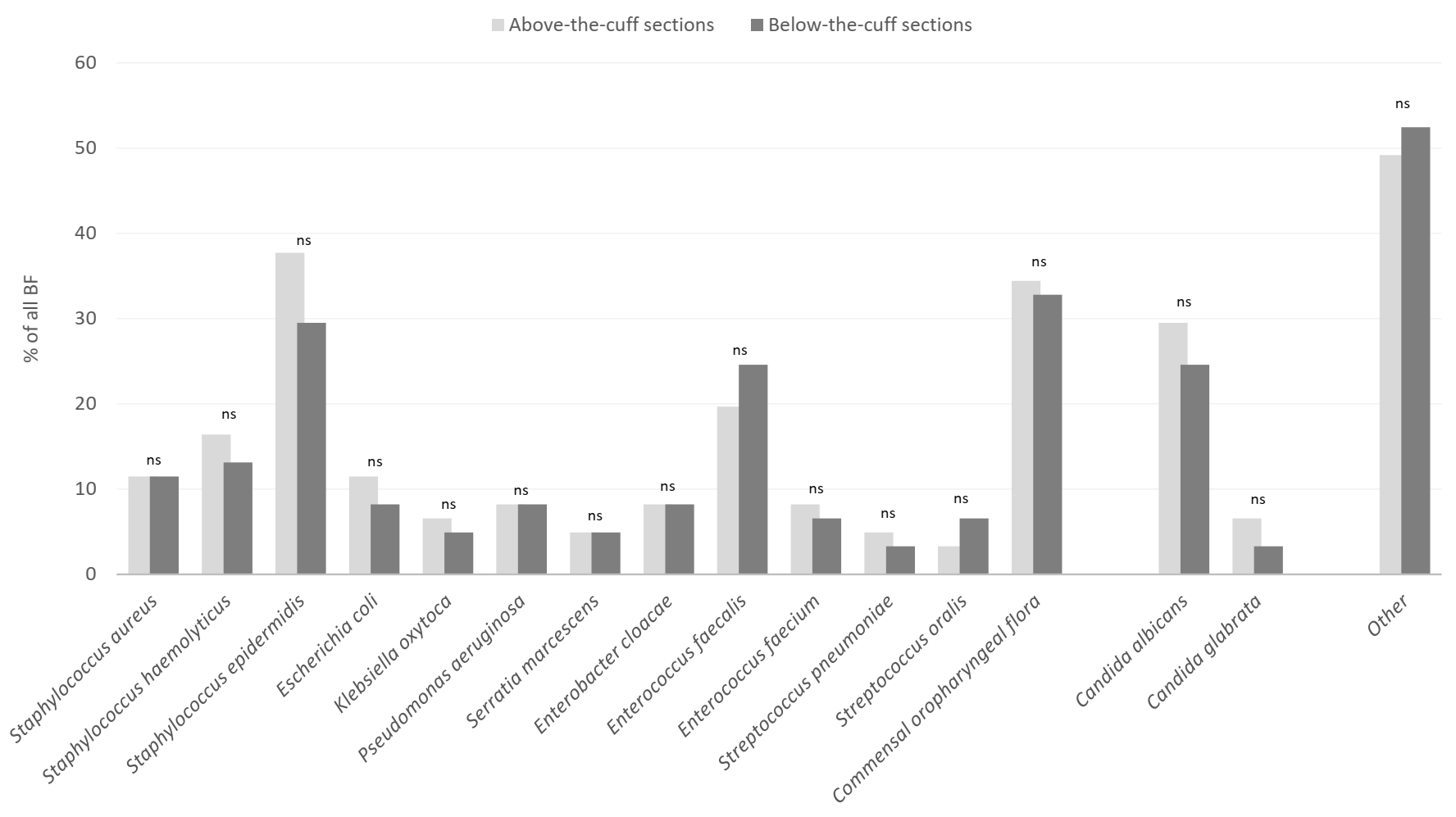


BF biofilm, ETT endotracheal tube, ns not significant

Other (microbiological species retrieved < 5%) : *Candida kefyr*, *Candida lusitaniae*, *Candida parapsilosis*, *Candida tropicalis*, *Citrobacter freundii, Enterobacter cancerogenus, Enterococcus durans, Klebsiella aerogenes*, *Klebsiella variicola*, *Lactobacillus gasseri, Lactobacillus paracasei, Lactobacillus rhamnosus*, *Morganella morganii, Neisseria mucosa, Propionibacterium acnes, Proteus mirabilis, Pseudomonas oryzihabitans*, *Raoultella ornithinolytica, Serratia rubidae, Staphylococcus hominis, Stenotrophomonas maltophilia*

***Figure S4***. Frequency of isolated microorganism in both biofilm and respiratory samples


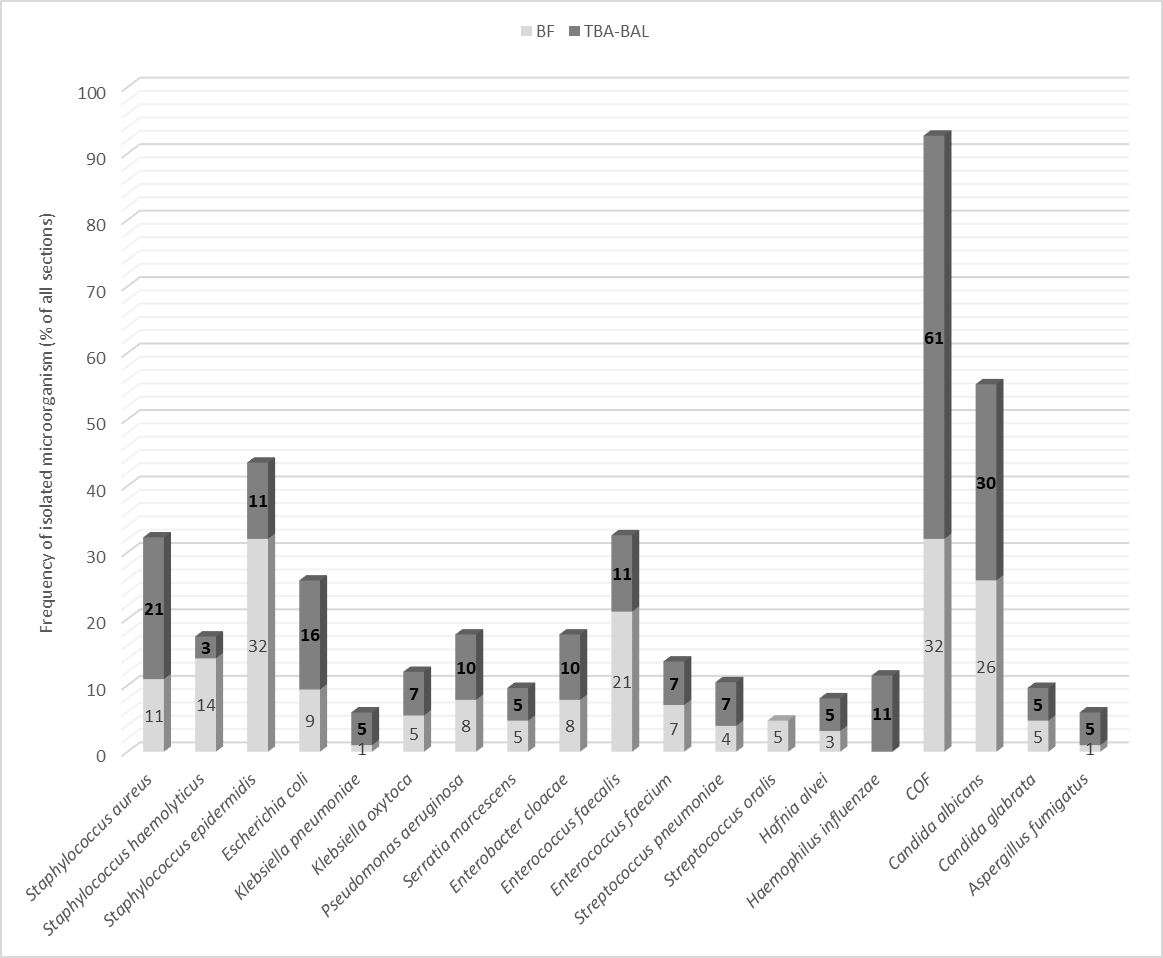


BAL bronchoalveolar lavage, BF biofilm, COF Commensal oropharyngeal flora, TBA tracheobronchial aspirate

***Figure S5*.** Microbiological characterization of ETT-deposited BF depending on the occurrence of VAP and COVID-19


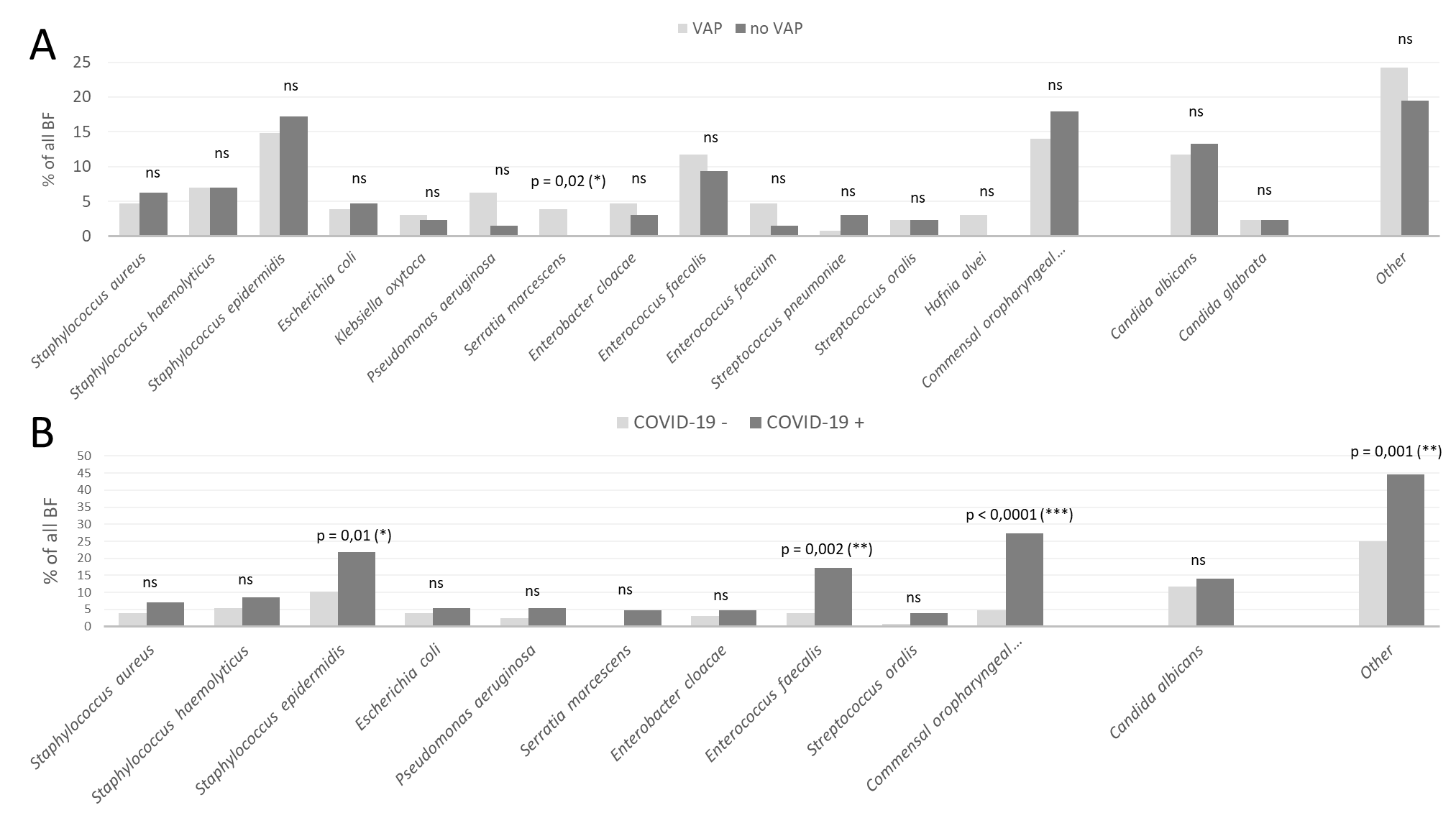


A. compared distribution of both bacterial and fungal species retrieved within ETT-deposited BF depending on the occurrence of VAP ; B. compared distribution of both bacterial and fungal species retrieved within ETT-deposited BF depending on the occurrence of COVID-19

BF biofilm, ETT endotracheal tube, ns not significant, VAP ventilator-associated pneumonia

Other (microbiological species retrieved < 5%) : *Candida kefyr*, *Candida lusitaniae*, *Candida parapsilosis*, *Candida tropicalis*, *Citrobacter freundii, Enterobacter cancerogenus, Enterococcus durans, Klebsiella aerogenes*, *Klebsiella variicola*, *Lactobacillus gasseri, Lactobacillus paracasei, Lactobacillus rhamnosus*, *Morganella morganii, Neisseria mucosa, Propionibacterium acnes, Proteus mirabilis, Pseudomonas oryzihabitans*, *Raoultella ornithinolytica, Serratia rubidae, Staphylococcus hominis, Stenotrophomonas maltophilia*
